# When insights based on ecological and cognitive theories to movement science converge—The case of creativity in sports

**DOI:** 10.3389/fpsyg.2022.959599

**Published:** 2022-08-17

**Authors:** Stephan Zahno, John van der Kamp

**Affiliations:** ^1^Institute of Sport Science, University of Bern, Bern, Switzerland; ^2^Department of Human Movement Science, Faculty of Behavioural and Movement Sciences, Vrije Universiteit Amsterdam, Amsterdam, Netherlands

**Keywords:** motor skill, motor learning, motor performance, creative actions, variability, adaptability

In sports, athletes strive for highly efficient and functional actions that make them competitive in their discipline. These actions are considered creative when they are also unforeseen, for example because they are new or rare in a specific situation. Sports practitioners increasingly recognize that creative actions provide a key competitive advantage (Glynn, 2013). In football, for example, in an attacking phase of play, a skilfully executed backheel pass can take the opponent by surprise and disrupt a well-organized defense to ultimately decide the game. Creative actions thus are not only highly functional but are also recognized as unconventional performances that make people marvel at sports. Accordingly, both sports practitioners and scientists are increasingly interested in understanding how creative actions come about and how they can be promoted. However, despite its intuitive appeal, creativity is a challenging topic; not the least because of the conceptual ambiguity surrounding it.

In recent years, we have independently conducted research into creativity from contrasting theoretical frameworks in movement science, ecological psychology (Orth et al., 2017; Withagen and van der Kamp, 2018) and cognitive theories of motor behavior (Zahno and Hossner, 2020). Interestingly, our approaches have resulted in converging conceptualizations of creativity in sports. This convergence may be surprising as ecological and cognitive theories to motor behavior are traditionally perceived to fiercely contest each other—a debate known as the “motor-action controversy” (Meijer and Roth, 1988). In this paper, we aim to highlight three converging insights on creativity in sports. We will further discuss how these insights contrast the current dominant approach to creativity and what they imply for ideas and practices that are widespread in sports. Finally, we reflect on the implications for the “motor-action controversy”.

Before focussing on creativity, we provide a brief background on the “motor-action controversy”. In the 1980s, ecological psychologists confronted the traditional “motor” theory, which held that movement control is mainly top-down determined by centrally stored representations (e.g., motor programs) that *pre*-scribe the details of the to-be produced movement pattern (e.g., Schmidt, 1975). Instead, ecological psychologists, using concepts of dynamic systems theory, proposed an “action” theory, where movement behavior is described in terms of lawful information-movement couplings (Warren, 1988). These lawful relationships emerge (or self-organize) within an individual's search to satisfy the dynamic constraints of the constituting person-environment relationship (Newell, 1986). In this view, control is as much a feature of the environment as of the person. Accordingly, learning entails an increased adaptive flexibility; that is, the emergence of movement pattern variability to maintain or expand a stable person-environment relationship (Davids et al., 2012). Learning is thus associated with degeneracy; meaning that similar stable movement behaviors can be achieved with different movement patterns. However, since the heydays of the “motor-action controversy”, fundamental conceptual re-orientations have been observed *within* cognitive or “motor” theories (see Hossner et al., 2020). Most notably, the idea of centrally stored motor programs that prescribe detailed movement patterns has been abandoned (Todorov and Jordan, 2002). Instead, James' (1890) ideomotor principle has been reinstated, expressing that behavior is controlled in terms of the anticipated effects (i.e., predicted perceptual consequences) of one's own actions (Prinz, 1997). Accordingly, in the theory of optimal feedback control (Todorov and Jordan, 2002), behavioral control is conceptualized as self-initiated transitions from current (perceived) situations to desired (perceived) situations. Thereby, it is a core feature of the theory that *not* each and every state dimension needs to be controlled. Rather, in the unfolding situation, motor commands are generated only if they essentially contribute to the maintenance or achievement of the desired situation. Learning, in this view, is thus conceptualized, *not* as acquiring centrally represented movement patterns, but as refining and expanding links between current (perceived) situations and the (perceived) effects in the environment of one's own actions. In cognitive theories, these links are conceptualized as forward models (Franklin and Wolpert, 2011). Or put differently, learning is also associated with degeneracy within this cognitive framework.

It is common for both sports practitioners and scientists to attribute creative actions, such as the backheel pass in football, to an athlete's ability to generate creative ideas (e.g., Memmert, 2015). This attribution has been adopted from traditional approaches in creativity research that are based on the concept of divergent thinking (Guilford, 1967). Divergent thinking tasks assess a participant's ability to generate multiple alternative ideas in response to a problem. Translating this into sports, this ability is assumed to enable the athlete to produce creative actions across a diverse range of sport-specific situations, and perhaps even beyond (Memmert and Roca, 2019). A corollary conjecture is that athletes can improve creativity with training programs dedicated to enhancing the ability to generate creative ideas (e.g., Memmert, 2021). Our recent research challenges this common view. We rather conceive creative actions as emerging from adaptations to momentary constraints, and thus as grounded in sensorimotor skill (Orth et al., 2017; Zahno and Hossner, 2020). Rather than enabled by a general, context-independent capacity for creativity that is active *before* the action, we argue that the “creative” of creative action arises *in* action and is thus always embedded in a sport-specific situation. But what do we understand as creative actions? This question leads to our first insight.

## Insight I: The “creative” in creative action reflects a judgement rather than being a property of the action or athlete

Creative actions refer to actions that are both functional (i.e., support task success) and considered novel, non-conventional (i.e., beyond typical standards) or rare in a particular context (cf. Runco and Jaeger, 2012). This implies that the “creative” in creative actions (or of creativity) is, in essence, an evaluative judgment of functional actions in terms of novelty, unconventionality, or rareness in one particular situation and not necessarily in another. This judgement of an action as being “creative” is inherently relative. It compares one action within a defined historical and social situation with other actions in the same situation (Westmeyer, 1998; Csikszentmihalyi, 1999). Most basically, this can be described as the statistical rareness of the action in that situation (Simonton, 2003; Caso and van der Kamp, 2020). Two consequences can be derived from this insight: (1) The same action can be highly creative in one context while being ordinary in another, just as the same action can be entirely novel at one point in time and become standard repertoire of a domain ever after. In other words, the “creative” in creative actions is not an inherent property of an action but is always defined relative to the situation in which it arises (Zahno and Hossner, 2020). Accordingly, we have shown in football that some environments (i.e., small-sided games) invite more and different creative actions than others (i.e., 11-aside) (Caso and van der Kamp, 2020). (2) The “creative” in creative action does not refer to some magical source or ability that forms the action. It is not something that athletes “possess” or “acquire”. Importantly, however, this does not imply that the athlete should be considered irrelevant. To the contrary, creative actions are grounded in the athlete's skill or adaptive flexibility (Ericsson, 1999). This brings us to the second insight.

## Insight II: Creative actions are grounded in athletes' sensorimotor skill rather than in an ability to generate ideas

Skilled athletes show a large, more variable movement repertoire. This enhances adaptive flexibility, allowing them to produce actions in ways that less skilled players cannot. Consequently, such actions are more likely to be statistically rare and thus creative. Accordingly, to promote creative actions, coaches should foster athletes' sensorimotor skill rather than their ability to generate ideas. We have tested this hypothesis in a recent field-based experiment in elite youth football (Zahno and Hossner, in press). Players who participated in football-specific divergent thinking training did indeed improve their capacity for creative idea generation. However, these improvements did not induce more creative actions on-field. In contrast, players who received motor skill training not only improved in functionality but performed more creative actions on-field. Beyond, in beginner football, Orangi et al. (2021) showed that a variable motor skill training, which aimed to channel players' search for adaptive movement patterns, resulted in more variable and creative movement behaviors than motor skill training that prescribed desired movement patterns. These results suggest that creative actions are not based on players' ability to generate ideas *per se*, but rather on what they *can* do *in* the situation. Consistent with both ecological and cognitive-ideomotor theories, this underlines that creative actions are grounded in sensorimotor skill, and particularly in the skilled athletes' adaptive flexibility to solve unfolding situations in multiple ways. This adaptiveness brings the person-environment relation to the forefront and leads to our third insight.

## Insight III: Creative actions are relational rather than a product of the individual alone

Dick Fosbury's revolutionary high jump technique won him the Olympics in 1968. A critical constraint in the creation of this novel technique was the replacement of the sand pit by crash mats in high-jump competitions. This shifted the boundaries of the task space for high jumping, allowing Fosbury to explore new task solutions such as landing on his back. Interestingly, Debbie Brill, a young athlete from Canada independently discovered the same technique around the same time. This shows that the invention of the Fosbury Flop cannot be understood as originating from Dick Fosbury solely but is co-constituted by and an adaption to a changing sport-specific context. Creative actions are thus always defined across the person-environment relationship. Consequently, creative actions cannot be trained as a de-contextualized ability. Rather, when designing practice, coaches should take sport-specific situations as a starting point and invite athletes to explore different ways of solving the situation; for example, by manipulating task constraints to make athletes adapt to changing constraints and thereby enhance variability of actions (Hristovski et al., 2011; Orth et al., 2017). Empirically, recent studies in football (Caso and van der Kamp, 2020; Orangi et al., 2021) and boxing (Orth et al., 2019) have confirmed that inducing a large variability of actions enhances the chance for creative actions to emerge.

To conclude: Our approaches to creativity in sports originate from traditionally opposed theoretical perspectives but converge in how they explicate creative actions and derive implications for practice. Creative actions are grounded in sensorimotor skill, wherein a large and variable movement repertoire associated with adaptive flexibility increases the likelihood for actions to arise that are recognized as “creative”. For sports practice, this suggests that creative actions are best promoted by motor skill training, especially when designing sport-specific environments that invite athletes to safely explore, discover and invent a rich repertoire of actions to solve movement problems (see e.g., Rasmussen et al., 2019).

This convergence, obviously, does not dissolve the “motor-action controversy”. Fundamental differences between “motor” and “action” theories remain. Arguably, the one that stands out is the ontology of internal mechanisms in “motor” theories vs. the sufficiency of the information-movement coupling in “action” theories for explaining movement behavior. From an applied perspective, however, instead of debating the veracity of the two theories, it seems more fruitful to recognize that, in the end, they are both models that aim to understand the reality of human movement behavior. And if distinct theories converge to a similar understanding, then perhaps we have increased our grip on that reality. Surely, this strengthens confidence in the practical recommendations that are derived—in this case, for sports. Intriguingly, and consistent with our current thinking, it may exactly be the variability in theoretical approaches that increases the likelihood of new conceptualizations that extend our understanding in a field of study. In this respect, we must cherish the “motor-action controversy”, rather than solving or—worse—ignoring it. That is, the interactions between the “motor” and “action” theories during the debate were an important impetus for theoretical developments. We thus believe that it is crucial to revive the cross talk between the theories particularly since open discussion and in-depth conceptual analysis of where the (sub)fields of the study of human movement behavior converge can benefit science as well as the practice of sports and beyond.

## Author contributions

SZ and JK contributed to conceptualization, background research, and draft work. All authors contributed to the article and approved the submitted version.

## Funding

Open access funding was provided by University Of Bern.

## Conflict of interest

The authors declare that the research was conducted in the absence of any commercial or financial relationships that could be construed as a potential conflict of interest.

## Publisher's note

All claims expressed in this article are solely those of the authors and do not necessarily represent those of their affiliated organizations, or those of the publisher, the editors and the reviewers. Any product that may be evaluated in this article, or claim that may be made by its manufacturer, is not guaranteed or endorsed by the publisher.
